# A Body Shape Index (ABSI) as a Variant of Conicity Index Not Affected by the Obesity Paradox: A Cross-Sectional Study Using Arterial Stiffness Parameter

**DOI:** 10.3390/jpm12122014

**Published:** 2022-12-05

**Authors:** Daiji Nagayama, Kentaro Fujishiro, Yasuhiro Watanabe, Takashi Yamaguchi, Kenji Suzuki, Atsuhito Saiki, Kohji Shirai

**Affiliations:** 1 Department of Internal Medicine, Nagayama Clinic, Oyama-City, Tochigi 323-0032, Japan; 2 Center of Diabetes, Endocrinology and Metabolism, Toho University, Sakura Medical Center, Sakura-City, Chiba 285-0841, Japan; 3 Research and Development Division, Japan Health Promotion Foundation, Shibuya-ku, Tokyo 150-0013, Japan; 4 Department of Internal Medicine, Mihama Hospital, Chiba-City, Chiba 261-0013, Japan

**Keywords:** a body shape index, conicity index, abdominal obesity index, obesity paradox, approximation, cardio-ankle vascular index

## Abstract

A body shape index (ABSI) is an abdominal obesity index developed based on epidemiological statistics and designed to correlate minimally with body mass index (BMI). We examined the approximation between ABSI and other abdominal obesity indices based on biophysical concepts. The cross-sectional data from 62,514 Japanese urban residents were analyzed. Body adiposity indices comprising BMI, waist circumference (WC), ABSI, conicity index (CI), waist-to-height ratio (WHtR), and WC/BMI ratio were examined. ABSI and CI more strongly correlated with age and arterial stiffness assessed by cardio-ankle vascular index (CAVI) compared to the other indices. The discriminative power for high CAVI (≥9.0) was the strongest for ABSI followed by CI and other indices, in that order. The range and distribution of WC corresponding to the cutoff of ABSI (0.0801), or CI (1.23) seemed reasonable. The correlation between ABSI and CI was the strongest compared to any other combination of indices. CI correlated moderately with BMI, whereas ABSI correlated minimally with BMI. ABSI correlates strongly and approximates closely with CI. Hence, ABSI may be considered to reflect the degree of body shape change from cylindricity to conicity and is currently the only abdominal obesity index not affected by the obesity paradox.

## 1. Introduction

Visceral adiposity promotes atherosclerosis directly or indirectly via metabolic disorders [1]. On the other hand, body mass index (BMI), an index commonly used to determine obesity, does not necessarily reflect visceral fat accumulation that contributes to vascular toxicity. In contrast, increased BMI is associated with improved vascular function, suggesting that BMI may mainly reflect vasoprotective body composition [2,3]. This is supported by the phenomenon of the obesity paradox in which increased BMI per se contributes to a favorable prognosis despite the presence of obesity-related metabolic disorders [4].

Recently, various abdominal obesity indices have been developed to assess visceral adiposity [5]. Waist circumference (WC) is now considered to be the gold standard of the abdominal obesity index and is also adopted in the diagnostic criteria for metabolic syndrome. However, WC is epidemiologically almost identical to BMI and does not necessarily reflect visceral fat accumulation, similar to BMI [6]. Consequently, a BMI-independent abdominal obesity index is needed to avoid the obesity paradox. A body shape index (ABSI) is an abdominal obesity index derived from a study of the United States National Health and Nutrition Examination Survey (NHANES) 1999–2004 mortality data [7]. This index is designed to be minimally associated with BMI and is calculated by dividing WC by an allometric regression of weight and height. However, the problem is that the ABSI formula includes a design based on epidemiological statistics, and its meaning is therefore difficult to understand. On the other hand, other abdominal obesity indices including WC, conicity index, waist-to-height ratio (WHtR), and WC/BMI ratio are based on biophysical concepts, and the meaning of the formulas is relatively easy to comprehend. Accordingly, translating ABSI into a biophysical concept may facilitate the use of ABSI in future studies of obesity-related disorders.

Arterial stiffness reflects the aging and loss of elasticity of blood vessels and is used as a predictor of cardiovascular disease (CVD) [8]. Cardio-ankle vascular index (CAVI), a systemic arterial stiffness parameter derived from stiffness parameter β and Bramwell–Hill’s equation, is generally measured using VaSera VS-series (Fukuda Denshi Co. Ltd., Tokyo, Japan) [9]. This parameter has been reported to be associated positively with the number of CVD risk factors, the severity of CVD, and future cardiovascular (CV) events. Appropriate therapeutic interventions to reduce CAVI are expected to contribute to the prevention of future CV events [10]. Additionally, various translational research linking the molecular mechanisms of arterial stiffness to physiological vascular function as assessed by CAVI has simultaneously been conducted [11]. Recently, we have demonstrated in a previous study that ABSI is more strongly associated with CAVI compared to other abdominal obesity indices [6]. However, the detailed relationship between abdominal obesity indices and systemic arterial stiffness has not yet been fully validated.

We conducted a cross-sectional study aiming to examine the approximation between ABSI and abdominal obesity indices based on biophysical concepts, comprising BMI, WC, conicity index, WHtR, and WC/BMI ratio. First, we compared the indices with respect to age-dependent changes and correlation with arterial stiffness. Next, we identified the cutoff value of each index for discriminating arterial stiffening (CAVI ≥ 9.0) and simulated how each index at its cutoff value actually relates to changes in body shape. Finally, the correlations between all combinations of the abdominal obesity indices were analyzed.

## 2. Results

### 2.1. Clinical and Biochemical Characteristics of Male and Female Participants

A total of 62,514 Japanese urban residents (female:male = 58.4:41.6; median age 42 years; median BMI 21.7 kg/m^2^) were studied in this cross-sectional study. Table 1 compares the clinical characteristics of male and female participants.

Compared with women, men had significantly higher BMI, WC, WHtR, blood pressure, fasting plasma glucose (FPG), and triglyceride (TG), and lower age, ABSI, WC/BMI ratio, and high-density lipoprotein cholesterol (HDL-C). Conicity index and CAVI showed little sex differences.

### 2.2. Correlation of Each Adiposity Index with Age or CAVI by Obesity Grade

A simple correlation analysis of the relationship of each body adiposity index with aging or systemic arterial stiffening was performed as shown in Table 2. Since BMI was speculated to affect body adiposity indices, the correlation was examined also for each BMI-based obesity grade.

Among the six indices, ABSI and conicity index correlated most strongly with age regardless of obesity grade, followed by WHtR. The index that showed the strongest correlation with CAVI was ABSI, followed by the conicity index. However, the degree of CAVI dependence was almost equal across all obesity grades. Taken together, ABSI and conicity index may be strong abdominal obesity indices for assessing the age-related progression of abdominal obesity and also arterial stiffening. On the other hand, BMI and WC showed a clearly weak correlation with age or CAVI. Especially, the correlation between BMI and CAVI was reversed from positive to negative when BMI ≥ 25 kg/m^2^.

### 2.3. Discriminatory Power of Each Adiposity Index for High CAVI (≥9.0)

Next, the discriminatory power of each body adiposity index for high CAVI was examined using ROC analysis as shown in Table 3.

Among the adiposity indices examined, ABSI and the conicity index had the highest discriminatory power for high CAVI, while BMI and WC had the lowest. In particular, the discriminatory power of ABSI was slightly higher than that of the conicity index (difference in C-statistics, *p* < 0.0001). The cutoff of each abdominal obesity index for predicting high CAVI was as follows: ABSI 0.0801, conicity index 1.23, WHtR 0.491, and WC/BMI ratio 0.0362. Incidentally, we have previously reported that ABSI of 0.080 is a sex-independent cutoff value that predicts not only high CAVI but also renal impairment (eGFR < 60 mL/min/1.73 m^2^) [12].

### 2.4. Waist Circumference Distribution Calculated from the Cutoff of Each Abdominal Obesity Index

**Figure 1:**
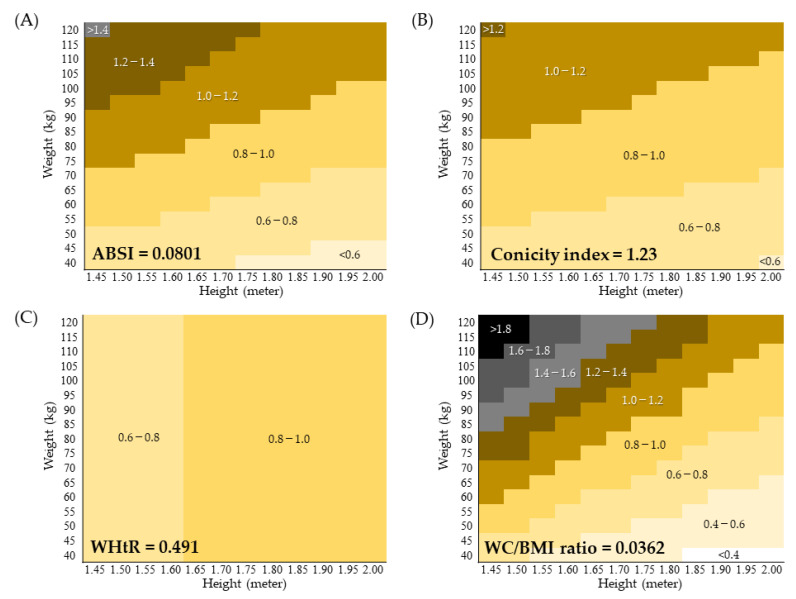
Waist circumference (m) matrix constructed from weight, height, and the cutoff of each abdominal obesity index. In a matrix, each cell that intersects a column (height) and a row (weight) is the WC calculated from that height and weight at the cutoff value. The waist circumferences are categorized by color into various levels. Cutoff values of (**A**) ABSI, (**B**) conicity index, (**C**) WHtR, and (**D**) WC/BMI ratio are shown in the matrices. ABSI, a body shape index; WHtR, waist-to-height ratio, WC, weight circumference; BMI, body mass index.

ABSI, conicity index, and WC/BMI ratio are calculated from WC, height, and weight (WHtR from WC and height). When the cutoff of an index is known, it is possible to calculate the WC for a given combination of weight and height at that cutoff value and construct a WC matrix as shown in Figure 1. In the matrix, each cell that intersects a column (height) and a row (weight) is the WC calculated from that height and weight and the cutoff value. Hence, the matrix provides a visual display of the range, distribution, and categorization of waist circumferences at the cutoff value.

In the present study, the median (interquartile range), minimum and maximum WC values for all subjects analyzed were 0.78 (0.72–0.85), 0.43, and 1.48 m, respectively. Considering this data, the range and distribution of WC for the cutoff of ABSI (Figure 1A) or conicity index (Figure 1B) seem reasonable. On the other hand, the distribution of WC for the WHtR cutoff (Figure 1C) was limited to a narrow range, while that for the WC/BMI ratio cutoff was too wide (Figure 1D). Note that the simulated WC greater than 1.6 m or less than 0.4 m in the WC/BMI ratio matrix suggest that this index does not reflect the real-world clinical situation.

### 2.5. Waist Circumference Calculator Chart Corresponding to Cutoff of ABSI

**Figure 2:**
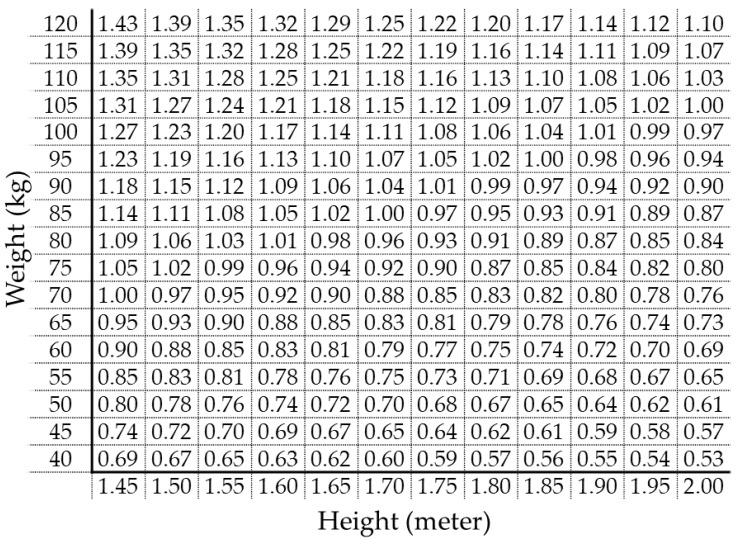
Waist circumference (m) chart for the ABSI cutoff at 0.0801. Colored distribution of waist circumference is shown in Figure 1A. An example of clinical use: If a person 1.70 m in height and 85 kg in weight has a waist circumference of 1.00 or higher, his/her ABSI is estimated to be 0.0801 or higher.

In daily clinical practice, a matrix chart may be a convenient tool to estimate whether a person has ABSI below or above the cutoff (0.0801), if his/her weight, height, and WC measurements are known. Figure 2 is the WC chart for ABSI 0.0801, which is the matrix of Figure 1A with WC values shown in all the cells. For example, if a person 1.70 m in height and 85 kg in weight has a WC of 1.00 or higher, his/her ABSI is estimated to be 0.0801 or higher, indicating abdominal obesity with the risk of arterial stiffening.

### 2.6. Correlation between Body Adiposity Indices

Finally, as shown in Table 4, a simple correlation analysis was performed to determine the BMI dependence of each abdominal obesity index as well as the approximation between the four abdominal obesity indices.

Abdominal obesity indices except ABSI were clearly correlated with BMI. In the combinations of abdominal obesity indices, ABSI and conicity index showed the strongest correlation (R_s_ = 0.909, 95% CI = 0.907–0.910). Regarding dependence on BMI, ABSI showed a very weak correlation with BMI (R_s_ = 0.063), whereas all the other abdominal obesity indices had a moderate to strong correlation with BMI. Comparing the R_s_ for all pairs of the four abdominal obesity indices, ABSI and conicity index showed the strongest correlation (R_s_ = 0.909, 95% CI = 0.907–0.910). The correlation between ABSI and the conicity index was slightly stronger in women (females vs. males R_s_ (95% CI): 0.929 (0.928–0.931) vs. 0.904 (0.902–0.907)) and was almost independent of obesity grade (R_s_: 0.984 for BMI < 20, 0.982 for BMI 20–24.9, 0.985 for BMI 25–29.9, 0.965 for BMI ≥ 30 kg/m^2^).

## 3. Discussion

This cross-sectional study conducted in middle-aged Japanese urban residents without a history of CVD revealed that among the abdominal obesity indices examined, ABSI and conicity index best reflect the age-related progression of abdominal obesity and systemic arterial stiffening. ABSI was associated with high CAVI slightly more strongly than the conicity index. The cutoffs for high CAVI were ABSI 0.0801 and conicity index 1.23. The WC distributions corresponding to the cutoffs of ABSI and conicity index appear to be valid. ABSI and conicity index showed the strongest correlation compared to any other combination of abdominal obesity indices, indicating the epidemiological approximation of the two indices. However, unlike ABSI, the conicity index also correlated with BMI, suggesting a concern that it may be influenced by the obesity paradox. 

To discuss the approximation between ABSI and the conicity index, we first outline the derivation of the formulas for the two indices. According to a previous report, ABSI was constructed by first performing linear least-squares regression on ln(WC) as a function of ln(Height) and ln(Weight) for a nonpregnant adult sample, with the following result [6]:

ln(WC) = (−2.589 ± 0.020) − (0.0814 ± 0.020) × ln(Height) + (0.6807 ± 0.0052) × ln(Weight)

Then, the obtained regression coefficients were approximated with ratios of small integers, yielding the following:

WC ∝ Height^−5/6^ × Weight^2/3^

When ABSI was defined to be proportional to the ratio of the actual WC to the WC estimated from the regression allometry, the following formula was obtained:

ABSI = WC × Height^5/6^ × Weight^−2/3^

In contrast to ABSI, the concept of a conicity index is based on biophysics, which assumes the human body is a cylinder [12]. This index is defined as the ratio of the measured WC divided by the reference WC, where the reference WC is the circumference (m) calculated from the height and weight. An increase in the conicity index implies the transformation from cylindricity to conicity (i.e., the progression of abdominal obesity). The conicity index has been reported to correlate strongly with the visceral fat area assessed by computed tomography (CT) [13]. In developing the conicity index, the following equation was first established [14]:

Weight = ρ × πR^2^ × Height, where ρ is human body density (=1050 kg/m^3^), R is the radius (m).

The circumference (m) is therefore expressed as follows:

2πR = 2 × √(π/ρ) × √(Weight/Height)

Since the conicity index is the ratio of WC to 2πR, the following formula of the conicity index is obtained:

Conicity index = WC/2πR = 9.174 × WC × Height^1/2^ × Weight^−1/2^

As shown above, ABSI and conicity index are distinguished by slight differences in the multipliers for height and weight. The two indices can be inter-converted as follows:

ABSI = 0.109 × BMI^−1/6^ × Conicity index

This conversion formula means that ABSI is easily obtained by correcting the conicity index by BMI. ABSI is derived from epidemiological statistics, while conicity index is derived from biophysical concept. It is interesting that the two indices with completely different origins approximate each other well. Considering the findings in the present study, ABSI may be regarded as a variant of the conicity index that is not affected by the obesity paradox.

We previously reported that weight reduction therapy with a formula diet improves CAVI in Japanese type 2 diabetic patients [15]. In that study, the change in visceral fat area assessed by CT independently contributed to the change in CAVI, whereas the change in weight did not. Unfortunately, abdominal obesity indices were not examined in that study, otherwise ABSI could have accurately demonstrated the involvement of visceral fat in vascular dysfunction, in the same manner as CT. On the other hand, abdominal obesity indices other than ABSI are influenced by weight change per se, and thus are expected to be less likely to reflect the change in visceral fat induced by weight reduction therapy.

ABSI accurately reflects visceral fat accumulation [16] and is suitable for screening individuals with CVD risk factors [17] and predicting CV mortality [18]. Rather than using BMI or WC alone, the use of ABSI in routine clinical practice may be greatly beneficial. Leone et al. reported that ABSI is an independent predictor for metabolic syndrome (MetS) in obese children and adolescents [19]. Indeed, adopting ABSI instead of WC in the diagnosis of MetS may enhance the predictive ability of high CAVI [12] and future renal function decline [20] in the general population. Further studies are needed to verify whether ABSI-lowering therapeutic interventions decrease CV events.

As shown in Table 1, the gender differences in metabolic parameters including blood pressure, FPG, and lipid parameters were observed. Nevertheless, we determined that these differences do not affect the conclusion of the present study, because there is no gender difference in the discriminatory power of ABSI for high CAVI. Furthermore, as noted above, the ABSI cutoff for increased arterial stiffness and renal impairment has been reported to be equal in both genders [12]. We, therefore, established the WC calculator that corresponds to the common ABSI cutoff for both genders (Figure 2). On the other hand, since WC, conicity index, and WHtR show stronger discriminatory power for high CAVI in women, it may be desirable to set these cutoffs separately for men and women.

The limitations of this study include the cross-sectional study design that does not allow the evaluation of a causal relationship. Moreover, our findings may not be generalized to other ethnic groups. In addition, the cutoffs of the abdominal obesity indices calculated in this study should be applied only to the middle-aged Japanese general population, because the cutoff for age-dependent factors is affected by the age distribution of the participants. Although only CAVI was employed in this study to determine the cutoff for the abdominal obesity indices, validation with other vascular parameters (i.e., flow-mediated dilation, pulse wave velocity, and carotid intima–media thickness) is also desired.

## 4. Materials and Methods

### 4.1. Subjects and Design

We performed a retrospective cross-sectional study on middle-aged Japanese urban residents who underwent health screening in Japan between April 2010 and March 2019. 

### 4.2. Data Collection and Methods of Measurement

The population-based sample used in the present analysis comprised 76,720 Japanese subjects residing in major cities nationwide. The subjects participated in the CVD and cancer screening program organized by the Japan Health Promotion Foundation. Participants were volunteers who were not paid and were not recruited for this study (unlike subjects of a clinical trial). 

Since CAVI, an arterial stiffness parameter used in this study, is modified by treatments for metabolic disorders and CVD, subjects taking any medication and/or having a history of heart disease or stroke, or treatment for hypertension, diabetes, nephritis, or gout were excluded. We finally enrolled 62,514 subjects.

All parameters were assessed using standardized methods. Height and weight were measured, and BMI (kg/m^2^) was calculated as follows: weight (kg) divided by square of height (m). WC (m) was measured horizontally at the height of the umbilicus, with the participant standing and arms hanging relaxed. 

For this study, we selected the abdominal obesity indices that have been reported to be useful in several major medical journals, and which can be calculated from height (m), weight (kg), and WC (m) as follows [14,21,22,23]:

A body shape index [21] = WC/(BMI2/3 × Height1/2) = WC × Height5/6 × Weight−2/3

Conicity index [14] = WC/{0.109 × √ (Weight/Height)} = 9.174 × WC × Height1/2 × Weight−1/2

Waist-to-height ratio [22] = WC × Height−1

WC/BMI ratio [23] = WC × Height × Weight−2

Blood was collected from the antecubital vein in the morning after 12 h of fasting to measure FPG, TG, and HDL-C. All the blood levels were measured by standard methods. 

### 4.3. Measurement of CAVI as an Arterial Stiffness Parameter

The principle and measurement methods for CAVI have been described previously [9]. CAVI was calculated according to the following formula: CAVI = a{(2ρ/ΔP) × ln(Ps/Pd)PWV^2^} + b, where Ps is systolic blood pressure; Pd is diastolic blood pressure; ΔP is Ps − Pd; ρ is blood density; PWV is cardio-ankle pulse wave velocity, and a and b are constants. Subjects with ankle-brachial indices lower than 0.90 were excluded, because patients with severe arterial occlusive diseases may give falsely low CAVI. We arbitrarily defined “high CAVI” as equal to or higher than 9.0 in all participants, corresponding substantially to the cutoff for the presence of coronary artery stenosis [10,24].

### 4.4. Statistical Analysis

The SPSS software (version 11.5, Chicago, IL, USA) was used for statistical processing. All data are expressed as median (interquartile range). Mann–Whitney U test was performed to examine sex differences in clinical variables. Spearman’s rank correlation coefficient was used to determine the relationship between clinical variables. In all comparisons, 2-sided *p* values < 0.05 were considered statistically significant. Alternatively, the difference between two variables was considered statistically significant if the 95% confidence intervals (CIs) for the correlation coefficients did not overlap. Sensitivity and specificity for predicting high CAVI were analyzed using conventional receiver operating characteristic (ROC) curves, and the cutoff was estimated by Youden’s J statistic. Based on the cutoff of each abdominal obesity index, WC corresponding to height and weight was calculated. For each selected abdominal obesity index, a WC matrix was constructed by calculating WC from wide ranges of height and weight and the cutoff value for that index.

## 5. Conclusions

In conclusion, ABSI correlates strongly and approximates closely with the conicity index, and their formulas are mathematically inter-convertible. Hence, ABSI may be considered to reflect the degree of body shape change from cylindricity to conicity and is currently the only abdominal obesity index not affected by the obesity paradox. This index may be most useful for discriminating malignant obesity that exerts vascular toxicity and is expected to be utilized in routine clinical practice.

## Figures and Tables

**Table 1:** Clinical and biochemical characteristics of male and female participants.

Variable	All Subjects	Males	Females	*p* Value *
Number of subjects	62,514	26,037	36,477	-
Age (years)	42 (34–54)	40 (33–51)	45 (36–55)	<0.001
Height (m)	1.62 (1.56–1.69)	1.71 (1.67–1.75)	1.57 (1.54–1.61)	<0.001
Body weight (kg)	57.2 (50.1–66.6)	66.9 (60.7–74.1)	51.6 (47.2–57.0)	<0.001
BMI (kg/m^2^)	21.7 (19.8–24.0)	23.0 (21.2–25.2)	20.8 (19.1–22.9)	<0.001
WC (m)	0.78 (0.72–0.85)	0.82 (0.76–0.89)	0.75 (0.70–0.82)	<0.001
ABSI	0.0785 (0.0757–0.0815)	0.0778 (0.0754–0.0803)	0.0791 (0.0760–0.0825)	<0.001
Conicity index	1.21 (1.16–1.26)	1.207 (1.16–1.25)	1.210 (1.15–1.26)	0.012
WHtR	0.480 (0.444–0.521)	0.484 (0.449–0.520)	0.477 (0.441–0.521)	<0.001
WC/BMI ratio	0.0358 (0.0342–0.0375)	0.0357 (0.0342–0.0372)	0.0360 (0.0342–0.0378)	<0.001
CAVI	7.3 (6.8–8.0)	7.3 (6.8–8.0)	7.3 (6.8–8.0)	<0.001
SBP (mmHg)	120 (111–130)	124 (116–132)	118 (108–128)	<0.001
DBP (mmHg)	70 (64–78)	74 (67–81)	68 (62–76)	<0.001
FPG (mg/dL)	84 (79–90)	86 (81–91)	82 (78–88)	<0.001
TG (mg/dL)	76 (54–113)	96 (67–146)	66 (49–93)	<0.001
HDL-C (mg/dL)	67 (56–80)	58 (49–69)	74 (63–86)	<0.001

Data are presented as median (interquartile range). * Analyzed by Mann–Whitney U test. BMI, body mass index; WC, waist circumference; ABSI, a body shape index; CAVI, cardio-ankle vascular index; WHtR, waist-to-height ratio; SBP, systolic blood pressure; DBP, diastolic blood pressure; FPG, fasting plasma glucose; TG, triglyceride; HDL-C, high-density lipoprotein cholesterol.

**Table 2:** Correlation of age or CAVI with each adiposity index by obesity grade.

	No. of Subjects		BMI	WC	ABSI	Conicity Index	WHtR	WC/BMI Ratio
**With Age**								
Total	62,514	R_s_	0.111	0.203	0.389	0.387	0.346	0.128
		95% CI	0.103–0.119	0.195–0.211	0.382–0.396	0.380–0.394	0.339–0.353	0.120–0.136
BMI < 20 kg/m^2^	17,570	R_s_	0.044	0.197	0.322	0.326	0.355	0.186
		95% CI	0.029–0.059	0.182–0.211	0.308–0.336	0.312–0.339	0.342–0.369	0.171–0.200
20 ≤ BMI < 25 kg/m^2^	33,695	R_s_	0.082	0.241	0.426	0.430	0.463	0.215
		95% CI	0.071–0.092	0.230–0.251	0.417–0.435	0.421–0.439	0.454–0.471	0.204–0.225
25 ≤ BMI < 30 kg/m^2^	9532	R_s_	−0.065	0.116	0.386	0.369	0.382	0.192
		95% CI	−0.085–−0.044	0.095–0.136	0.369–0.404	0.351–0.387	0.365–0.400	0.172–0.211
30 kg/m^2^ ≤ BMI	1717	R_s_	−0.139	0.002	0.296	0.266	0.278	0.132
		95% CI	−0.187–−0.090	−0.048–0.051	0.250–0.340	0.220–0.311	0.232–0.323	0.084–0.181
**With CAVI**								
Total	62,514	R_s_	0.034	0.149	0.332	0.305	0.217	0.197
		95% CI	0.026–0.042	0.141–0.156	0.325–0.340	0.298–0.312	0.209–0.225	0.189–0.205
BMI < 20 kg/m^2^	17,570	R_s_	0.001	0.193	0.291	0.287	0.263	0.221
		95% CI	−0.014–0.016	0.179–0.208	0.277–0.305	0.273–0.301	0.249–0.278	0.207–0.236
20 ≤ BMI < 25 kg/m^2^	33,695	R_s_	0.058	0.250	0.364	0.365	0.342	0.250
		95% CI	0.047–0.069	0.239–0.260	0.354–0.373	0.356–0.375	0.332–0.352	0.240–0.261
25 ≤ BMI < 30 kg/m^2^	9532	R_s_	-0.106	0.114	0.309	0.286	0.229	0.229
		95% CI	−0.127–−0.086	0.094–0.135	0.290–0.328	0.267–0.305	0.210–0.249	0.209–0.248
30 kg/m^2^ ≤ BMI	1717	R_s_	−0.183	0.037	0.260	0.221	0.138	0.223
		95% CI	−0.230–−0.134	−0.012–0.086	0.213–0.305	0.173–0.267	0.089–0.186	0.175–0.269

R_s_, Spearman’s rank correlation coefficient; 95% CI, 95% confidence interval; CAVI, cardio-ankle vascular index; BMI, body mass index; WC, waist circumference; ABSI, a body shape index; WHtR, waist-to-height ratio.

**Table 3:** Discriminatory power of body adiposity indices for high CAVI (≥ 9.0).

Index	Cutoff	C-Statistic	95% CI	*p* Value
BMI (kg/m^2^)	21.6	0.521	0.513–0.530	<0.001
WC (m)	0.799	0.597	0.589–0.605	<0.001
ABSI	0.0801	0.714	0.706–0.721	<0.001
Conicity index	1.23	0.700	0.692–0.707	<0.001
WHtR	0.491	0.645	0.637–0.653	<0.001
WC/BMI ratio	0.0362	0.627	0.619–0.636	<0.001

Discriminatory power, receiver operating characteristics analysis for discriminating the probability of high CAVI; 95% CI, 95% confidence interval; CAVI, cardio-ankle vascular index; BMI, body mass index; WC, waist circumference; ABSI, a body shape index; WHtR, waist-to-height ratio.

**Table 4:** Correlation between body adiposity indices.

Combination of Indices	Rs	95% CI	*p* Value
BMI vs. WC	0.870	0.868–0.872	<0.001
BMI vs. ABSI	0.063	0.054–0.071	<0.001
BMI vs. Conicity index	0.440	0.433–0.446	<0.001
BMI vs. WC/BMI ratio	−0.551	−0.557–−0.545	<0.001
BMI vs. WHtR	0.812	0.809–0.814	<0.001
WC vs. ABSI	0.465	0.458–0.471	<0.001
WC vs. Conicity index	0.766	0.763–0.769	<0.001
WC vs. WC/BMI	−0.107	−0.115–−0.099	<0.001
WC vs. WHtR	0.893	0.892–0.895	<0.001
ABSI vs. Conicity index	0.909	0.907–0.910	<0.001
ABSI vs. WC/BMI ratio	0.674	0.669–0.678	<0.001
ABSI vs. WHtR	0.553	0.547–0.558	<0.001
Conicity index vs. WC/BMI ratio	0.372	0.365–0.379	<0.001
Conicity index vs. WHtR	0.827	0.824–0.829	<0.001
WC/BMI ratio vs. WHtR	−0.166	−0.174–−0.158	<0.001

R_s_, Spearman’s rank correlation coefficient; 95% CI, 95% confidence interval; BMI, body mass index; WC, waist circumference; ABSI, a body shape index; WHtR, waist-to-height ratio.

## Data Availability

The data that support the findings of this study are not publicly available because they contain information that could compromise the privacy of research participants.
